# Epidemiological features of BLV natural infection

**DOI:** 10.1186/1742-4690-11-S1-P45

**Published:** 2014-01-07

**Authors:** Marina Lomonaco, Irene Alvarez, Cecilia Martínez, Natalia Porta, Ramiro Merlini, Hugo Carignano, Gerónimo Gutiérrez, Karina Trono

**Affiliations:** 1 Instituto de Virologia, INTA, Castelar, Argentina; 2 Instituto de Genetica, INTA, Castelar, Argentina

## 

Dairy farms are heavily infected with Bovine Leukemia Virus (BLV) in Argentina and many other countries, where a control strategy should be design based on the behavior of natural. We conducted a series of studies with the aim to better understand the epidemiology of BLV. Infected new born calves were present in 3 studied farms (8.3%-11%). Proviral load (PVL) was very low in 9/10 new born analyzed calves but rapidly augmented during the first months of age. Cross-sectional studies showed that the rate of high PVL between seroreactors raised together with the prevalence, from 20.2% at 8 months of age to 44.4% in 26 months heifers, with similar levels to adult lactating cows of the same farm. Low fluctuation of blood PVL was observed on animals with naturally-acquired infections. We also observed 10 seroconversions between young heifers with an initial elevation of PVl. The presence of provirus in colostrum was significantly correlated with blood PVL (p≤0.0001). Provirus in milk was detectable in bulk tank (17.2%) and individual samples (40.4%). Colostrum of individual cows showed different provirus/antibodies dual profiles that permit to speculate about different infective/protective potential among infected animals. These findings suggest animals would be exposed to the infective challenge since a very young age. Consequently, it must be control as soon as possible after birth. The main focus should be put on the new-born infected calves that could play the role of main propagators together with the putative oral exposition through colostrum and milk.

